# Evaluation of the performance of register data as indicators for dairy herds with high lameness prevalence

**DOI:** 10.1186/s13028-019-0484-y

**Published:** 2019-10-21

**Authors:** Nina Dam Otten, Nils Toft, Peter Thorup Thomsen, Hans Houe

**Affiliations:** 1 Department of Veterinary and Animal Sciences, Faculty of Health and Medical Sciences, University of Copenhagen, Grønnegårdsvej 8, 1870 Frederiksberg C, Denmark; 2 Section for Epidemiology, National Veterinary Institute, Technical University of Denmark, Kemitorvet, 2800 Kgs Lyngby, Denmark; 3 Department of Animal Science, Aarhus University, P. O. Box 50, 8830 Tjele, Denmark; 4 Present Address: IQinAbox ApS, Måløv Byvej 229, 2760 Måløv, Denmark

**Keywords:** Dairy cattle, Indicators, Lameness, Register data, ROC

## Abstract

**Background:**

The modern dairy industry routinely generates data on production and disease. Therefore, the use of these cheap and at times even “free” data to predict a given state of welfare in a cost-effective manner is evaluated in the present study. Such register data could potentially be used in the identification of herds at risk of having animal welfare problems. The present study evaluated the diagnostic performance of four routinely registered indicators for identifying herds with high lameness prevalence among 40 Danish dairy herds. Indicators were extracted as within-herd annual means for a one-year period for cow mortality, bulk milk somatic cell count, proportion of lean cows at slaughter and the standard deviation (SD) of age at first calving. The target condition “high lameness prevalence” was defined as a within-herd prevalence of lame cows of  ≥ 16% (third quartile). Diagnostic performance was evaluated by constructing and analysing Receiver Operating Characteristic curves and their area under the curve (AUC) for single indicators and indicator combinations. Sensitivity (Se) and specificity (Sp) of the indicators were assessed at the optimal cut-off based on data and compared to a set of predefined cut-off levels (national annual means or 90-percentile).

**Results:**

Cow mortality had the highest AUC (0.76), while adding the three other indicators to the model did not yield significant increase in AUC. Cow mortality and SD of age at first calving had highest Se (100%, 95% confidence interval (CI): 72–100%), while highest Sp was found for the proportion of lean cows at slaughter (83%, 95% CI: 66–93%). The highest differential positive rate (DPR = 0.53) optimizing both Se and Sp was found for cow mortality. Optimal cut-off points were lower than the presently used pre-defined cut-offs.

**Conclusions:**

The selected register-based indicators proved to be able to identify herds with high lameness prevalences. Optimized cut-offs improved the predictive ability and should therefore be preferred in official control schemes.

## Background

Increased public focus on animal welfare has led to the implementation of various welfare assurance schemes for commercial use, especially for cattle and pig herds. Parallel to these industry-based welfare schemes, authorities also conduct animal welfare controls. In order to obtain valid national estimates of the national welfare level of e.g. dairy herds, on-farm assessments would have to be performed in all herds. Such assessments would require direct animal-based measures of the clinical and behavioural state of animals, as these are perceived to be closest to the true state of welfare. However, such data are very costly to retrieve due to investigator training and calibration and the actual time needed for herd visits. Hence, a more targeted approach is needed. This approach could be used for pre-screening of herds at risk of having welfare problems, subsequently reducing the number of herds visited. For this purpose, register data could be utilised, as direct consequences of clinical manifestations are reflected in e.g. milk production and reproductive results available through register data. The Danish welfare control programme uses register-based indicators to identify livestock herds at risk of welfare problems based on a set of risk parameters from the national databases. This initial screening is followed by a control visit by the authorities in selected herds. The initial screening is based on certain cut-offs for the given parameters, but there is a need to investigate how sensitive these cut-offs are and how optimized cut-offs would perform instead, not only for the official selection of herds but also in other welfare aspects such as commercial welfare assurance schemes.

Within modern livestock production, vast amounts of data are generated and routinely recorded in databases. Data like disease recordings and production results are of great value for epidemiological research and have traditionally been used in e.g. investigating risk factors. Over the past decades, non-specific routine registrations (i.e. secondary data) have also become of interest in so-called syndromic surveillance schemes [1–5]. These surveillance schemes have been used for different purposes in different species such as disease outbreak prediction in cattle [6, 7], pigs [8] and horses [9]. Likewise, various register-based indicators such as treatment records have previously been used to predict more distinct clinical manifestations in dairy cattle. Milk production data, e.g. milk yield, bulk milk somatic cell count (BMSCC) and fat/protein ratio have been studied for associations with several outcome measures like mortality [10, 11], lameness related diseases [12, 13] and metabolic disorders [14, 15]. A review by deVries et al. [16] investigating associations between register-based variables and welfare indicators from the Welfare Quality^®^ assessment protocol found contradicting reports on the associations between variables related to productivity and welfare indicators. That led to the conclusion, that even though numerous associations between register-based indicators and direct clinical and behavioural conditions exist, the ability of register-based indicators to estimate animal welfare is not fully understood. These indicators are often limited to uncover only a few aspects of the multi-dimensional complex of animal welfare.

In Denmark, all livestock herds are required to register birth, death and movement of animals to the Central Husbandry Register (CHR). All treatments with prescription drugs performed by either a veterinarian or the farmers must be reported to the national database VetStat. Additionally, an industry database, the Danish Cattle Database (DCD), compiles data from the official databases, the milk recording scheme, breeding associations, laboratory findings and abattoirs. These data have been used to assess animal welfare in dairy herds with different welfare definitions. Otten et al. [17] investigated register-based indicators for violations of animal welfare legislation as would be detected by official animal welfare control. The reported increasing probability of violations with increasing variation (standard deviation [SD]) in milk yield between cows in first lactation if herds had a BMSCC > 250,000 cells/mL and less than 25 veterinary treatments/100 cow years, emphasize the association between management and animal welfare. Animal welfare experts often refer to lameness as ‘the most important’ animal welfare measure [18–20] in modern dairy production. Not only due to the painful nature of most locomotor disorders [21–23] but also due to the subsequent impact of decreased fertility and longevity [24–27]. Hence, the use of register data as a screening tool might prove valuable for monitoring certain welfare aspects such as direct clinical measures like lameness.

In order to explore the opportunities for predicting a direct physiological state on a given day, the present study seeks to combine data from different sources in order to see if register data are able to predict high lameness prevalences. Therefore, the objectives of the present study were to investigate the predictive ability of different register data indicators to identify dairy herds with high lameness prevalence and compare presently predefined cut-offs with optimized cut-offs of the given indicators.

## Methods

For the purpose of this paper, lameness data gathered in 40 Danish dairy herds in a previous study by Thomsen et al. [28] were used to establish the target condition of high within-herd lameness prevalence (for details on herd and cow sampling please see [28]).

### Clinical protocol—target condition

The lameness score used was a five point ordinal scale described in [25] ranging from a score 1 for non-lame cows to a score 5 for severely lame cows. In the present study cows with a score 4 or 5 were classified as “lame”. The overall mean within-herd lameness prevalence across all herds was 12.9 ± 9.88% (SD) with a median of 11%. The obtained mean herd level prevalence was dichotomized using the third quartile as a cut-off. This led to a classification of herds into either having a low lameness level for herds having a mean prevalence of lame cows < 16%; or a high lameness level for herds with a prevalence ≥ 16%.

### Register-based indicators

A literature review on register-based indicators for impaired animal welfare in dairy cows was performed. Findings in previous Nordic studies [29–31] lead to the choice of four indicators representing different aspects of a dairy cow’s lifespan i.e. from entry to exit of the milk production period. The indicators cow mortality, BMSCC, proportion of lean cows at slaughter defined as cows with fat score 1 according to the EU Beef Carcase Classification Scheme and SD of age at first calving were chosen for the present analysis and were extracted from the DCD for the year 2004 as annual means per 100 cow years.

Indicators were assessed in two different models: a *data*-*driven model* evaluating indicators measured as continuous variables and a *predefined cut*-*off model* based on dichotomization of indicators based on predefined cut-offs. National means for the year 2004 (according to the Danish Knowledge Centre for Agriculture and the dairy industry) were used as predefined cut-offs for the indicators cow mortality, BMSCC and SD of age at first calving. The cut-off for the indicator lean cows at slaughter was not based on the national mean, as this was as low as 15% compared to the variable mean of 24% in the present sample. Hence, the cut-off was chosen to reflect the national 90th-percentile.

### Statistical analyses

All associations between the outcome (low or high lameness) and the indicators as continuous variables were assessed in univariable and multivariable screening in logistic models using the glm function in *R* [32]. Additionally, indicators were also assessed as dichotomized variables according to the predefined cut-offs by testing their associations with low or high lameness prevalence using McNemar tests. Correlations between explanatory variables were evaluated by means of Spearman´s correlation coefficient.

Sensitivity (Se) and specificity (Sp) estimates and their 95% confidence intervals (95% CI) at the optimal cut-off points were determined. Se was defined as the fraction of herds with an indicator level above the given cut-off among herds with high lameness level, i.e. with a prevalence of lame cows ≥ 16%. Sp was defined as the fraction of herds with an indicator level below cut-off among herds with low lameness level (< 16%).

The optimal cut-offs maximizing the differential positive rates (DPR = Se + Sp−1) of each indicator were identified by analysing Receiver Operating Characteristic curves (ROC). The predictive ability was quantified by assessing the area under the curve (AUC) [33]. All ROC analyses were made using the *R*-package pROC [34]. All indicators were assessed individually followed by an assessment of different indicator combinations. Model selection was based on comparing the Akaike Information Criterion (AIC) (significance level of *P* < 0.05) of the given models. Finally, the differences in AUC between models were assessed using the rcomp function.

## Results

A descriptive summary of the indicators is given in Table 1. Amongst the continuous indicators only cow mortality (*P* = 0.04) and BMSCC (*P* = 0.05) showed significant associations with high lameness prevalences. The McNemar tests revealed significant associations between high lameness prevalences and mortality (*P *= 0.002), BMSCC (*P *< 0.001) and lean cows at slaughter (*P *< 0.001) while SD of age at first calving was not significantly associated with high lameness prevalences (*P* = 0.45). Mortality was significantly correlated to BMSCC (*P* = 0.03) and SD of age at first calving (*P* = 0.05).

**Table 1:** Descriptive statistics for four register-based indicators for discriminating between lameness prevalences ≥ 16% (High, n = 10) and < 16% (Low, n = 30) in 40 Danish dairy herds

	Mean		P-value*	Median	SD	Q1	Q3	Max
Indicator	Low	High		Low/High	Low/High	Low/High	Low/High	Low/High
Cow mortality^a^	4.6	7.3	0.04	3.6/5.0	2.9/3.9	0.0/4.9	7.3/9.7	10.2/16.1
Bulk milk SCC^b^	204	241	0.05	196/226	49/48	163/213	235/276	327/318
Lean cows^c^	24.1	25.5	0.8	24.4/23.9	14.7/15.6	11.9/13.3	33.3/41.3	59.5/43.6
SD age at first calving^d^	2.5	2.6	0.6	2.3/2.3	0.7/0.8	2.2/2.2	2.9/2.3	3.9/4.6

*P-values derived from the univariable screening for the differences in means between the two groups of lameness prevalence (high ≥ 16%, low < 16%)

^a^Annual mean cow mortality rate

^b^Bulkmilk somatic cell count × 1000 cells/mL

^c^Proportion of lean cows at slaughter out of total number slaughtered per herd (%)

^d^Standard deviation (SD) of age at first calving in months

Cut-offs maximizing the DPR are given in Table 2 together with the predefined cut-offs. The cut-offs for the indicators identified by the data-driven optimization approach were lower than the predefined cut-off except for the proportion of lean cows at slaughter.

**Table 2:** Comparison of cut-offs used in the dichotomization of indicators of high lameness prevalence in 40 Danish dairy herds

Variables	Cut-off	
	Pre-defined	Optimized
Cow mortality^a^ (%)	5.7	3.6
Bulk milk SCC^b^ (×1000 cells/mL)	245	214
Lean cows at slaughter^c^ (%)	40	10
SD age at first calving^d^ (months)	2.4	2.0

^a^Cow mortality = annual mean cow mortality rate per 100 cow years

^b^Bulk milk SCC = annual mean bulk milk somatic cell count based on monthly or bimonthly recordings

^c^Lean cows = percent of cows with fat score 1 according to the EU Beef Carcase Classification

^d^SD age at first calving = annual mean standard deviation

**Fig. 1:**
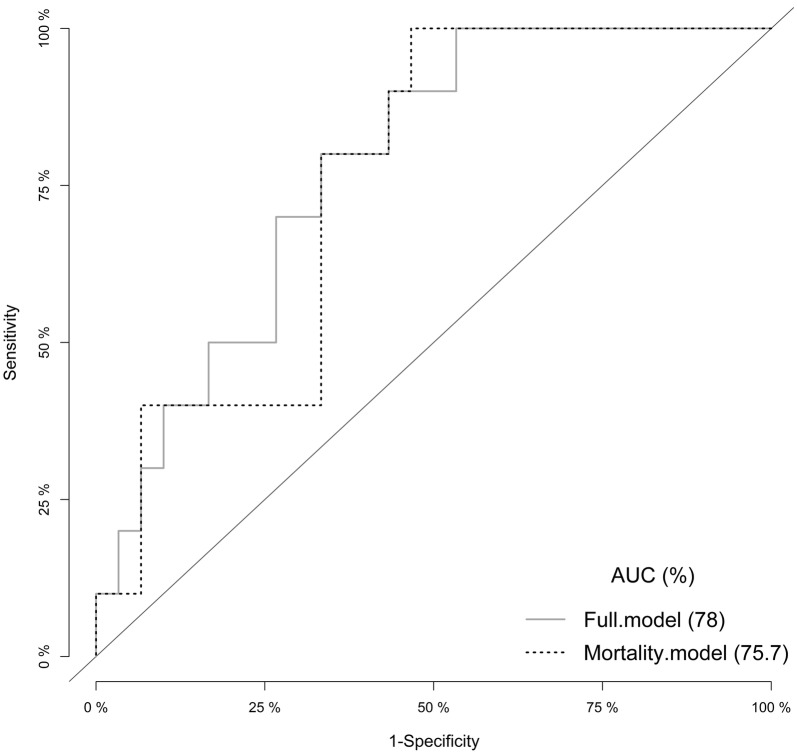
Receiver operating characteristic (ROC) plot of the indicator cow mortality quantifying the diagnostic potential to an area under the curve (AUC) of 76% (dashed line) compared to the full model including all four explanatory variables with an AUC of 78% (grey line)

Individual evaluation of the continuous indicators showed cow mortality having the highest AUC of 0.76, followed by BMSCC with an AUC of 0.73 (Table 3). Furthermore, only the AUC´s for cow mortality and BMSCC were significantly different from 0.5 (the random AUC of a test with no information). Comparison of the classification of case herds based on either predefined or data-driven cut-offs for maximizing the AUC, Se and Sp are shown in Fig. 1. In practice, this would mean that if herds were to be selected for animal welfare control based on the predefined cut-offs, four truly positive herds would be overlooked, while the number of false positives would be lower compared to data-driven approach.

**Table 3:** Model area under the curve (AUC) compared to the area under a random and non-informative receiver operating characteristic (ROC) curve (AUC = 0.5)

Indicator	Predefined cut-offs					Optimized cut-offs					
	Se (%) [95% CI]	Sp (%) [95% CI]	DPR^a^	PPV [95% CI]	NPV [95% CI]	Se (%) [95% CI]	Sp (%) [95% CI]	DPR	PPV [95% CI]	NPV [95% CI]	AUC(P*)*
Cow mortality^b^	40 [12;74]	67 [47;83]	0.07	29 [8;58]	77 [56;91]	100 [69;100]	67 [47;83]	0.67	39 [20;59]	100 [77;100]	0.76 (0.001)
BMSCC^c^	40 [12;74]	77 [58;90]	0.13	36 [11;69]	79 [60;92]	70 [35;93]	67 [47;83]	0.47	41 [18;67]	87 [66;97]	0.73 (0.01)
Lean cows^d^	40 [12;74]	83 [65;94]	0.23	44 [14;79]	81 [63;93]	90 [56;100]	20 [8;39]	0.10	27 [13;46]	86 [42;100]	0.53 (0.82)
SD age 1^st^ calving^e^	40 [12;74]	60 [41;77]	0	25 [7;52]	75 [53;90]	80 [44;98]	23 [10;42]	0.03	26 [12;45]	78 [40;97]	0.50 (0.95)

Sensitivity (Se), specificity (Sp) and predictive values (positive predictive values = PPV, negative predictive values = NPV) with 95% confidence intervals for indicators assessed at optimised and predefined cut-offs for the identification of high lameness prevalences (≥ 16%)

^a^DPR = Differential positive rates = Se + Sp –1 as a measure of test Se and Sp strength in combination

^b^Annual mean cow mortality rate

^c^Bulk milk somatic cell count × 1000 cells/mL

^d^Proportion of lean cows at slaughter out of total number slaughtered per herd (%)

^e^Standard deviation (SD)

**Fig. 2:**
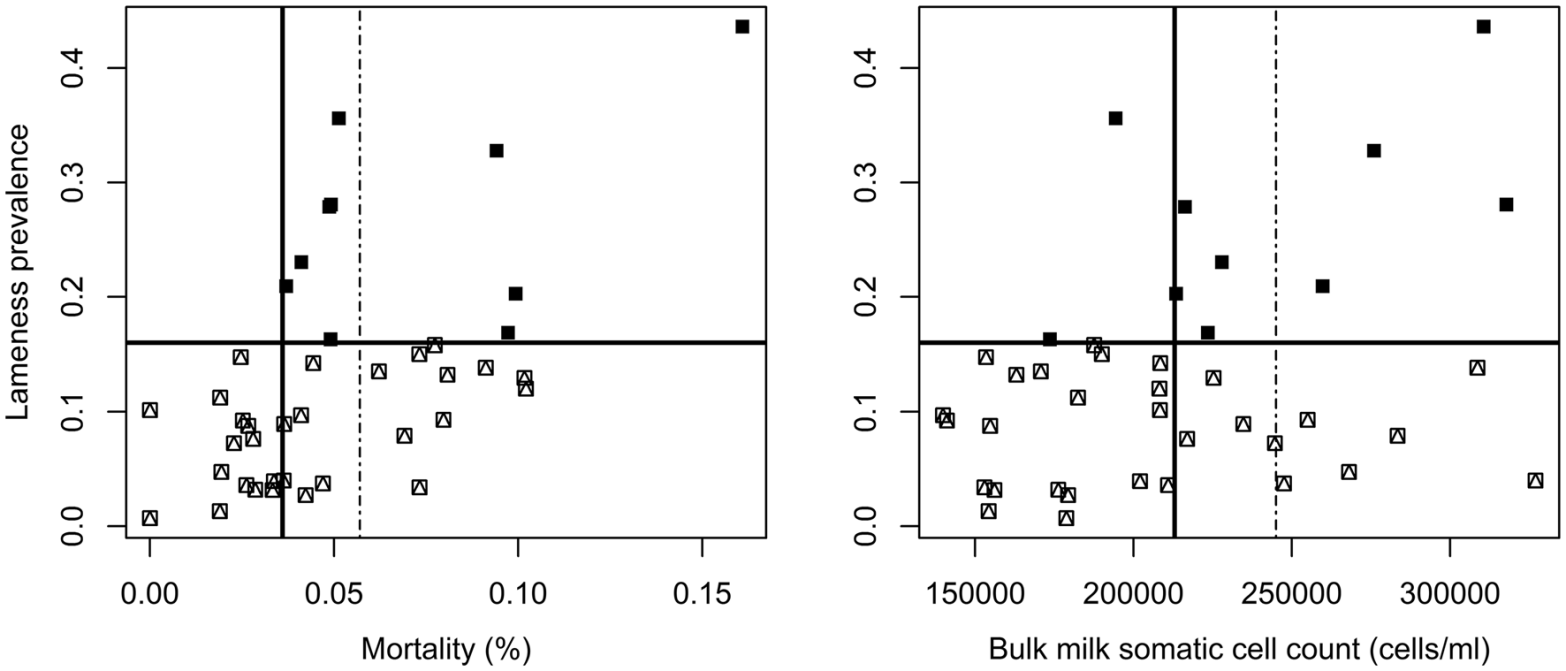
Plots of indicators (cow mortality and bulk milk somatic cell count) with predefined cut-offs (dashed vertical line) and maximized DPR (solid vertical line) against the lameness prevalence for 40 herds with cut-off (Q3 16%, horizontal black line)(▪herds with high levels of lameness,  herds with low level of lameness)

Highest Se was found for cow mortality (100%, 95% CI: 69–100%) and lean cows (90%, 95% CI: 56–100%), but with fairly low corresponding Sp (mortality 67%, 95% CI: 47–83%; lean cows 20%, 95% CI: 8–39%) (Table 3). Highest Sp was found for BMSCC and mortality (67%, 95% CI: 47–83%). Models evaluating indicators based on predefined cut-offs and the combination of cow mortality, BMSCC and lean cows achieved the highest AUC (0.71). Model parameters are shown in Table 4. Combining the variables yielded only a slight and non-significant improvement of the AUC (0.78) (Table 5 and Fig. 2).

**Table 4:** Parameter estimates, standard error (SE), P-values, Akaike Information Criteria (AIC) for univariable logistic regression models assessing the associations between high lameness prevalence and four indicators at predefined and optimized cut-offs

Indicator	Cut-off	Estimate	SE	P	AIC
Mortality	Predefined	−0.29	0.75	0.7	48.84
	Optimized	−19.1	2874.13	0.99	38.65
BMSCC	Predefined	−0.78	0.78	0.31	47.99
	Optimized	−1.54	0.79	0.05*	44.85
Lean cows	Predefined	0.13	0.73	0.86	48.95
	Optimized	−0.81	1.14	0.48	48.42
SD age at 1st calving	Predefined	0.31 × 10^−17^	0.0074	1.00	48.99
	Optimized	−0.2	0.9	0.83	48.94

**Table 5:** Results of stepwise addition of indicators given by the area under the curve (AUC), model fit by Akaike Information Criterion (AIC) and test for significant increase in model AUC (P-value) compared to the random curve

Indicators	AUC	P-value
Random curve	0.5	–
Cow mortality	0.76	0.03
Cow mortality + BMSCC	0.76	0.18
Cow mortality + BMSCC + lean cows at slaughter	0.78	0.76
Cow mortality + BMSCC + lean cows at slaughter + SD age at first calving	0.78	0.65

Indicators: Cow mortality = annual mean mortality rate per 100 cow years; BMSCC = bulk milk somatic cell count; lean cows at slaughter = proportion of cows per herd with a fat score 1 at slaughter; SD age at first calving = standard deviation of age at first calving

## Discussion

The present study shows that selected register based secondary data have a predictive ability to discriminate between high and low prevalences of lameness. Best combinations of Se and Sp were found for cow mortality (Se 100%, Sp 67%) and BMSCC (Se 70%, Sp 67%). For the two other indicators, Sp was high but at the expense of a low Se. The data-driven cut-offs with maximized DPR were lower than the predefined cut-offs. This shows that optimal cut-offs are dependent on the sample population, hence, extrapolation to the general population should only be made with great care. Lameness prevalences found in the present study corresponded well to previous Danish studies reporting herd level prevalences of severely lame cows ranging from 5–30% in cross-sectional studies [35–37] depending on the lameness definition. Animal welfare expert elicitation on the impact of lameness on animal welfare resulted in herd level prevalences of 9.3% [38] and 15% [20] as thresholds for unacceptable animal welfare, comparable to the chosen cut-off in the present study (16%).

Despite the general need for a multi-factorial approach in terms of assessing animal welfare, the annual Danish risk-based welfare surveillance scheme only aims at targeting livestock herds within the worst 5% range for a very limited set of indicators. Although it might be speculated that targeting a wider range of indicators covering more aspects of a dairy cow’s life like health, productivity and management would increase the sensitivity of the risk-based identification, the findings of the present study of mortality being the most potent indicator are in alignment with the current official identification scheme. Our findings emphasize the challenge of choosing the right thresholds, as the optimal cut-off for cow mortality was markedly lower than even the national means. Since the combinations of the remaining three indicators only yielded a significant difference from the random ROC curve when put in combination with cow mortality, none of these indicators proved to be better predictors than random chance; leaving mortality as the most potent indicator for high lameness prevalences. This association between cow mortality and lameness under Danish settings was expected, since locomotor disorders are the primary reason for 40% of all cases of euthanasia among Danish dairy cows [26], a finding also in alignment with other studies [24, 39–41]. The significant association between BMSCC and lameness found in the present study is in line with Peeler et al. [42] but in contrast to other studies looking at associations between BMSCC and lameness [43, 44] or mastitis and sole ulcers [45]. Adding the indicators BMSCC, lean cows at slaughter and SD in age at first calving in a stepwise manner to the mortality model did not improve the AUC and model quality (i.e. AIC). Nonetheless, the prediction model containing all four indicators might prove useful in other study samples. The present study used a small sample size of 40 herds yielding large CIs for the estimates and making ROC curves jagged, but still indicators showed acceptable predictive performance. However, the wide CIs imply a larger error margin, which could be decreased by increasing the sample size.

The generally lower DPR values for predefined cut-offs illustrate the pit-falls of using means or norms. Furthermore, it became obvious how essential it is to establish whether the chosen indicator should be used as an indicator and hence be used with optimal cut-offs rather than being investigated as a welfare problem per se with predefined thresholds. When using register-based indicators, it should be decided whether the variable is used solely as an indicator or whether it is assessed as a problematic condition itself, which often might be the case in the official risk-based control scheme. In case of cow mortality, this would mean that the herd specific cow mortality could be used to predict high lameness in herds based on optimized cut-offs. On the other hand, if predefined cut-offs are assigned to the herd specific mortality, it could be assessed as a problematic condition itself and not as an indicator. Therefore, the question, whether the given indicator is a risk factor for the outcome of interest or just another problem in itself, should be answered first.

Traditionally, risk-based surveillance or targeted surveillance focus on risk factors for given diseases leading to a more focused sampling of “high-risk populations” [46]. For this purpose, the quantitative measure needs to be converted into qualitative measures, a process leading to a general loss of information and to an unwanted loss in test sensitivity. Nonetheless, this conversion is essential in order to develop the first step in the risk-based surveillance scheme i.e. the identification of hazards and to stratify the population into subgroups. On the other hand, as the risk-based surveillance schemes act like initial screening tests, a high Se is needed [47], at least from the risk manager’s point of view. The subjects (herds/farm managers) being investigated would benefit from a highly specific model–avoiding false incrimination of herds with truly low lameness prevalence, although a subsequent control visit would reveal the mistake.

In order to identify herds at risk of having a given state, i.e. acceptable/unacceptable welfare or high/low lameness prevalence on a given day, certain cut-offs are established, which is rather challenging by the means of secondary incidence data. In order to mimic the official risk-based selection of herds, incidence data were restricted to cover only one year to obtain annual means of indicators for 2004. This caused differences in time periods before and after herd visits between herds, which may introduce biases e.g. due to seasonal effects. A systematic collection of register data with a fixed period before and after herd visits may have improved the predictive ability of our register data indicators. Otten et al. [31] investigated three different time periods showing differences in indicator combinations in models for each time period. If the accuracy of the current surveillance system should be improved, optimal cut-offs should be used for the risk-based sampling. However, in order to enhance the accuracy of the indicators, the general prevalence of impaired welfare should be investigated in a large-scale cross-sectional study. Furthermore, optimized cut-offs could also result in a higher number of farms initially being identified as ‘problem farms’ with an associated risk of challenging the implementation of such a surveillance due to operational constraints such as limited personnel and time.

Register-based indicators have been investigated in other surveillance settings, e.g. evaluated as naïve Bayesian classifiers to give updated probabilities of a given outcome of interest, e.g. of emerging diseases in animal populations [1, 48, 49]. However, before considering distinct models based on no-gold standard methods like latent class analysis or Bayesian methods, further investigations should be done to evaluate the effects of different time periods on the indicator performance.

## Conclusions

The present study shows that the quantitative assessment of register data can be used as a screening tool for direct cross-sectional measures. Nonetheless, the present findings highlight the need for evaluating indicators and predictive models as diagnostic tests for the given case definition in order to determine their predictive performance prior to implementation in surveillance schemes. In the case of high lameness prevalence, cow mortality proved to be the strongest indicator with a high sensitivity. Adding BMSCC, lean cows at slaughter and SD in age at first calving only yielded a slight improvement in specificity. Optimized cut-offs enhanced model accuracy and should be preferred in official control schemes. Finally, the purpose of using register data needs to be clear, as outcomes will vary whether these data are used as indicators or as cut-offs for a given problem. Further investigations evaluating cut-offs for register-based indicators within different strata of the target population are needed, as there may be large variations in cut-offs between e.g. organic and conventional herds or large, intensive loose-housed dairy systems compared to small, extensive tie-stall herds.

## Data Availability

The datasets used and/or analysed during the current study are available from the corresponding author on reasonable request.
